# Development of a Simplified Radiation-Induced Emulsion Graft Polymerization Method and Its Application to the Fabrication of a Heavy Metal Adsorbent

**DOI:** 10.3390/polym11081373

**Published:** 2019-08-20

**Authors:** Masaaki Omichi, Yuji Ueki, Noriaki Seko, Yasunari Maekawa

**Affiliations:** Department of Advanced Functional Materials Research, Takasaki Advanced Radiation Research Institute, Quantum Beam Science Research Directorate, National Institutes for Quantum and Radiological Science and Technology (QST), 1233 Watanuki-machi, Takasaki, Gunma 370-1292, Japan

**Keywords:** SREG, emulsion graft polymerization, dissolved oxygen, metal adsorbent, glycidyl methacrylate

## Abstract

A simplified radiation-induced emulsion graft polymerization (SREG) method is proposed. This method involves a convenient and easy degassing process of a monomer solution using a commercially available sealed glass jar. A loaded weight on the lid of the jar was used to control the jar’s internal pressure as the degassing of the monomer solution took place using a vacuum pump. The degassing method was highly reproducible, resulting from no bumping of the monomer solution. The initial grafting velocity was proportional to the absorbed doses of pre-irradiation between 5 and 20 kGy. This result indicates that dissolved oxygen was sufficiently eliminated from the monomer solution at such a level where the remaining oxygen had little effect on the grafting reaction at a dose of 5 kGy. The method was then applied to the fabrication of a heavy metal adsorbent that possessed a sufficient adsorption capacity of Co(II) ions. The SREG method is applicable to the fabrication of a wide variety of functional graft polymers because high-dose-rate gamma-ray radiation and expensive experimental equipment are not necessary.

## 1. Introduction

Radiation-induced graft polymerization is used for several industrial-scale biomedical and environmental materials because radiation grafting changes only the surface of the polymer without any impact on trunk polymers [1,2,3,4,5,6,7,8]. In general, there are two types of irradiation methods in graft polymerization: the simultaneous and the pre-irradiation methods [8,9,10,11]. In the simultaneous irradiation method, the polymer substrate is irradiated in the presence of a monomer. This method is simple and easy for polymer surface modification. However, one drawback is that it generates a large quantity of ungrafted homopolymers that are inseparable from the grafted polymer. In contrast, the pre-irradiation method relies on the polymer substrate being activated by irradiation in the absence of the monomer, and the activated polymer subsequently reacts with the monomer. Although this method is more complicated and very susceptible to dissolved oxygen in the monomer solution, compared with the simultaneous irradiation method, very little ungrafted homopolymer is formed, and it is possible to suppress the undesirable consumption of the monomer (monomer loss). The pre-irradiation method, therefore, is considered to be the more practically useful method.

We and colleagues previously reported an emulsion graft polymerization procedure using the pre-irradiation method, in which the monomer was emulsified by the surfactant in an aqueous system instead of in an organic solvent [12,13,14,15,16,17,18]. The process was remarkable in that it required a lower radiation dose, a decreased monomer concentration, and a shorter reaction period than the normal graft polymerization process, which uses organic solvents. Although the densely grafted product cannot be fabricated by the normal method at low-dose pre-irradiation, the emulsion graft polymerization method can achieve a sufficient degree of grafting, even when a low total dose irradiation (10 kGy) is used. The surfactant likely promotes the attack of the monomer molecule to the surface of the polymer substrate.

At low doses of pre-irradiation, oxygen dissolved in the monomer solution has a relatively significant effect on the graft polymerization reaction. The radicals generated in the crystalline phase in trunk polymers by irradiation are relatively stable in the atmosphere. However, when the irradiated polymer substrate is immersed in the monomer solution, the radicals rapidly react with dissolved oxygen instead of the monomer molecule. On a laboratory scale, using a gas/vacuum manifold is an effective way of reducing the amount of oxygen dissolved in solution, although the technique requires a skilled researcher. On an industrial scale for the mass production of graft polymers, the conventional graft polymerization method using a gas/vacuum manifold is not practical.

In practical use, the rolled nonwoven fabric is irradiated by gamma-rays at doses above 20 kGy, in which gamma-rays can penetrate deeply into the internal regions, and then graft polymerization is carried out under bubbling with nitrogen gas to eliminate the dissolved oxygen [19]. However, the nitrogen bubbles that adhere to the nonwoven fabrics decrease the surface area of the activated fabric that is attacked by the monomer molecules and inhibit uniform graft polymerization. This results in a decrease in both the degree of grafting and reproducibility. Moreover, elimination of oxygen cannot be accomplished completely by nitrogen bubbling. Without using a gas/vacuum manifold, it is difficult to reduce dissolved oxygen from the monomer solution below the concentration at which the graft polymerization is not inhibited. Graft polymerization under atmospheric pressure via bubbling nitrogen gas through the monomer solution does not work when the pre-irradiation dose is under 10 kGy.

It is also possible to eliminate dissolved oxygen in the monomer solution by using a vacuum pump. However, on releasing the vacuum, oxygen redissolves into the monomer solution. Furthermore, bumping of the monomer solution often occurs because it is difficult to control the speed of the pump. The emulsion graft polymerization method, especially, has the disadvantage of foaming because the monomer solution contains a surfactant. A careful vacuum degassing method is required to prevent the monomer solution from overflowing the reaction vessel. The development of a more convenient and industry-friendly graft polymerization method that proceeds with only low-dose pre-irradiation is expected to resolve these problems and enhance the value of the technology.

In this paper, we report a simplified radiation-induced emulsion graft polymerization (SREG) method that involves an easy procedure to sufficiently remove dissolved oxygen from the monomer solution, as well as its application to the fabrication of a heavy metal adsorbent.

## 2. Materials and Methods

### 2.1. Materials

Polyethylene-coated polypropylene (PE/PP) nonwoven fabric, which was kindly supplied by Kurashiki Textile Manufacturing Co., Ltd. (Osaka, Japan), was used as a trunk polymer for SREG. The average diameter of the PE/PP fabric was 13 μm. Glycidyl methacrylate (GMA) was purchased from Tokyo Kasei Chemical Industry Co., Ltd. (Tokyo, Japan). Polyoxyethylene sorbitan monolaurate (Tween 20) and cobalt(II) chloride were purchased from Kanto Chemical Co., Inc. (Tokyo, Japan). All the other reagents and solvents were purchased from Wako Pure Chemical (Osaka, Japan). All the chemicals were used as received without further purification.

### 2.2. SREG Method

PE/PP fabrics (size: 3 × 6 cm; average weight: 110 mg) were packed into a gas barrier bag. After the gas barrier bag was sealed under vacuum, the PE/PP fabrics were irradiated using a gamma-ray source at −79 °C (dry-ice). The total doses were controlled within a range of 5–20 kGy (10 kGy/h). The irradiated PE/PP fabrics were then stored in a −80 °C freezer until use. The monomer emulsion of 5 wt% GMA was prepared by adding GMA to a 0.5 wt% Tween 20 aqueous solution that was then stirred at room temperature for 5 min. After bubbling with nitrogen to displace dissolved oxygen in the GMA emulsion, 120 mL of the de-aerated GMA emulsion was poured into a sealed glass jar (WE-975, Weck, Germany) that contained the irradiated PE/PP fabric. The lid was closed, and a weight of 400 g was placed on the sealed jar’s lid. Degassing of the monomer solution (GMA emulsion) was carried out in a vacuum desiccator using an oil rotary vacuum pump (GLD-136C, ULVAC, Kanagawa, Japan) for 15 min. The pressure in the vacuum desiccator was monitored using a Pirani vacuum gauge (GP-1G/WP-01, ULVAC, Kanagawa, Japan) with a range of 0.4–2700 Pa. The grafting reaction was carried out by keeping the sealed glass jar in a 40 °C water bath. The reaction times were varied between 1 and 3 h. Residual monomers and homopolymers were removed by washing the resulting GMA-grafted PE/PP (PE/PP-*g*-GMA) fabrics with water and methanol. The amount of GMA grafted onto the PE/PP fabrics was expressed as the degree of grafting (*Dg* (%)), and *Dg* was calculated using the following equation:

(1) $$\mathrm{Degree}\;\mathrm{of}\;\mathrm{grafting}:Dg\;\left(\%\right)=\frac{\left(W_{1}-W_{0}\right)}{W_{0}}\times 100$$

where *W*_0_ and *W*_1_ are the dry weights of the PE/PP fabrics before and after grafting, respectively.

To confirm the GMA graft chain, thermogravimetric analysis (TGA) was performed using a TG-DTA6200 instrument (Seiko Instruments Inc., Chiba, Japan) at a heating rate of 10 °C/min under nitrogen flow (50 mL/min).

### 2.3. Introduction of Iminodiacetic Groups onto PE/PP-g-GMA Fabrics

To introduce the groups onto PE/PP-*g*-GMA, the PE/PP-*g*-GMA fabrics were treated with 0.5 M disodium iminodiacetate (IDA) in a 1:1 *v/v* ethanol/water solution at 70 °C for 24 h [20]. The resulting IDA-GMA-grafted PE/PP (PE/PP-*g*-GMA-IDA) fabric was washed with water and methanol to remove unreacted disodium iminodiacetate. The IDA group density of the fibrous metal adsorbent and the extent of the epoxy group conversion into IDA were defined as follows [21]:

(2) $$\mathrm{IDA}\;\mathrm{group}\;\mathrm{density}\;\left(\mathrm{mmol}-\mathrm{IDA}\;\mathrm{group}/g-\mathrm{adsorbent}\right)=\frac{\left(W_{2}-W_{1}\right)}{177\times W_{2}}\times 1000$$

(3) $$\mathrm{Conversion}\;\left(\%\right)=\;\frac{\left(W_{2}-W_{1}\right)\times 142}{\left(W_{1}-W_{0}\right)\times 177}\times 100$$

where *W*_2_ is the weight of the PE/PP-*g*-GMA-IDA fabric.

To confirm the identity of the reaction products, the PE/PP, PE/PP-*g*-GMA, and PE/PP-*g*-GMA-IDA fabrics were characterized using Fourier transform infrared attenuated total reflectance (FTIR-ATR) spectroscopy (spectrophotometer, Perkin-Elmer Frontier, Perkin-Elmer, Yokohama, Japan) in the range 4000–500 cm^−1^.

### 2.4. Heavy Metal Adsorption Test

Cobalt (Co) ions were used as a model for harmful heavy metal ions. The cobalt adsorption ability of the PE/PP-*g*-GMA-IDA fabric was evaluated through a batch adsorption test. In the batch test, a 1-cm diameter of PE/PP-*g*-GMA-IDA fabric (20 mg) was prepared by punching, and the fabric was dipped in 10 mL of 50 ppm cobalt solution (CoCl_2_, pH = 5.8) for 6 h at 25 °C. Solutions (50 μL) were sampled at 5, 15, 30, 45, 60, 90, 120, 180, 240, and 360 min. The sampling solutions were diluted 30-fold with 0.1 M HNO_3_ solution. The concentration of Co ion in the filtered sampling solutions was measured using inductively coupled plasma optical emission spectrometry (ICP-OES; Optima 8300, Perkin-Elmer Japan, Yokohama, Japan).

## 3. Results and Discussion

### 3.1. Application of the SREG Method to GMA Emulsion Graft Polymerization

**Scheme 1:**
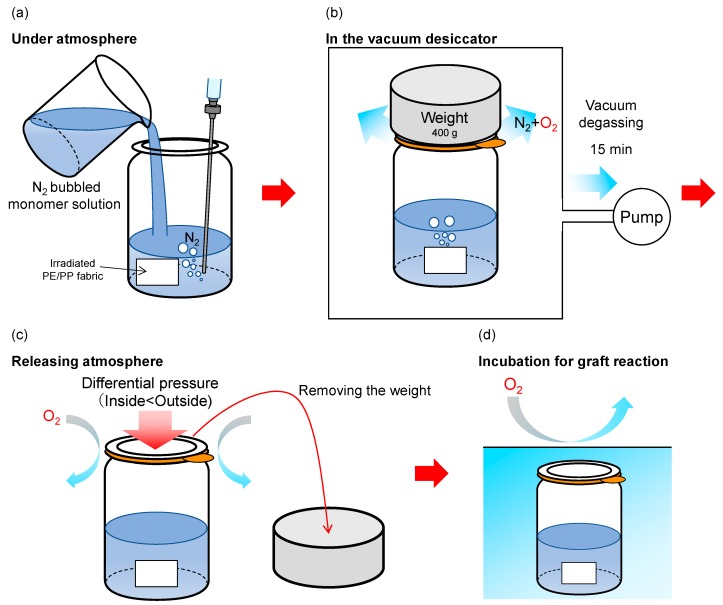
Schematic image of a simplified radiation-induced emulsion graft polymerization (SREG).

To solve the problem concerning the dissolved oxygen during emulsion graft polymerization as mentioned in “Introduction,” we introduced a new method, SREG, as shown in Scheme 1. GMA was chosen as a grafting agent, as it could be easily converted to structures having various functions [21,22,23,24,25,26,27]. The process is as follows: (a) The preirradiated PE/PP fabric was set in the commercially available sealed glass jar, made up of the glass lid, the rubber packing, and the jar itself. At ambient atmosphere, the de-aerated monomer emulsion was poured into the glass jar with nitrogen bubbling and then sealed. (b) After the lid was closed, a weight (400 g) was placed on top of the sealed gas jar, and degassing of the monomer solution was carried out in a vacuum desiccator. Dissolved oxygen in the monomer solution was then eliminated through the small space between the lid and the jar. (c) On releasing the desiccator’s vacuum, the loaded weight caused a pressure difference between the internal and external pressures. This pressure difference automatically maintained the vacuum state in the sealed gas jar and prevented atmospheric oxygen from re-entering the monomer solution. The loaded weight was removed from the lid. (d) The sealed glass jar was then held at a constant temperature in a water bath, and the graft reaction proceeded without oxygen.

**Figure 1:**
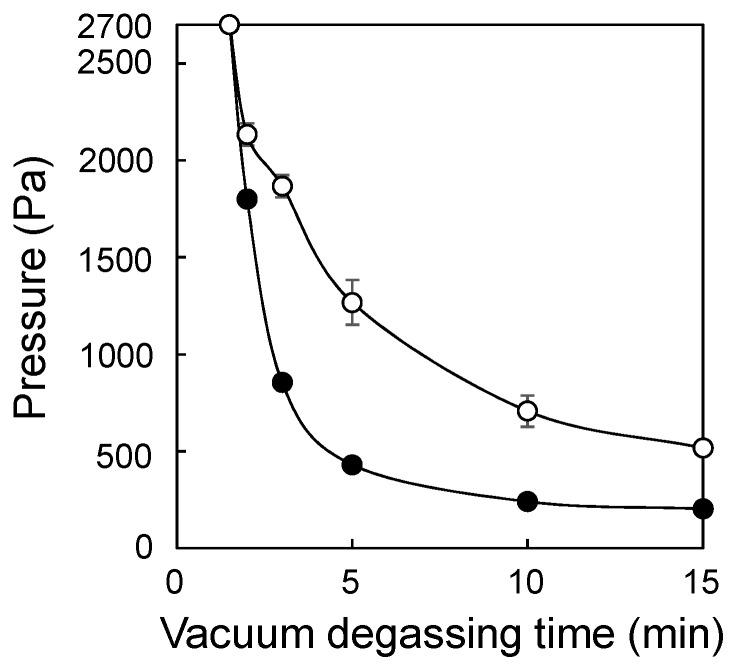
Relationship between pressure in the vacuum desiccator and vacuum degassing time. The sealed glass jar contained monomer solution (○) or did not contain monomer solution (●). Error bars indicate standard deviation.

The main role of the weight was to control the pressure in the sealed glass jar. If the atmospheric pressure in the jar suddenly decreased below the vapor pressure of the solution, bumping will occur. The weight allowed the pressure inside the jar to remain near the vapor pressure of the solvent, thus preventing bumping while allowing dissolved oxygen to be eliminated. Furthermore, evaporation of the solvent was negligible during vacuum degassing because evaporation of the solvent caused a drop in temperature, which in turn caused the vapor pressure to decrease. The vapor pressure of water was 1706, 2333, and 3173 Pa, at 15, 20, and 25 °C, respectively, and the pressure in the vacuum desiccator dropped to 200 Pa using the vacuum pump (Figure 1).

**Figure 2:**
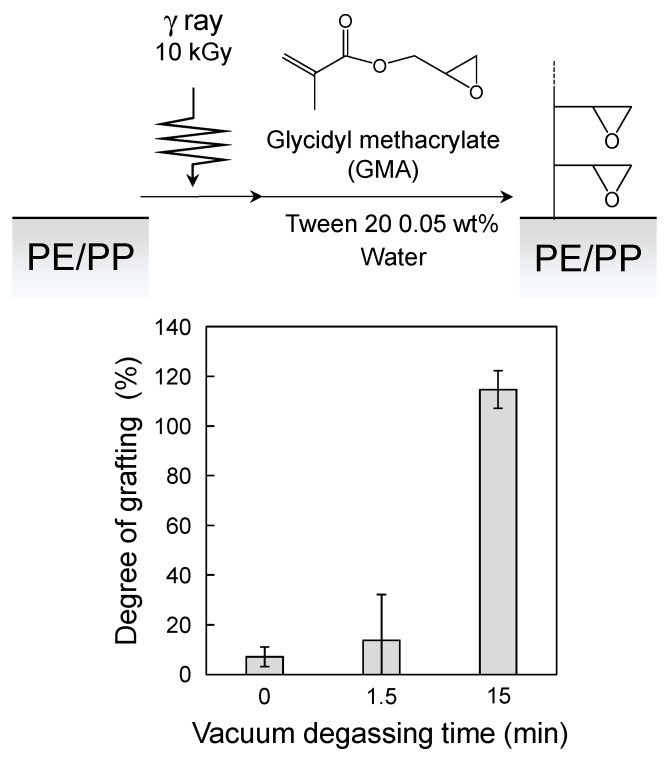
Relationship between degree of grafting and vacuum degassing time at 10 kGy. Error bars indicate standard deviation.

Considering these facts, the 400 g weight (the total weight, including the lid, was 445 g, area 28.3 cm^2^), corresponding to 1540 Pa, was chosen. When the pressure in the desiccator reached 2700 Pa, degassing in the monomer solution visibly started. Vigorous degassing was observed at 2000 Pa, and the pressure in the sealed glass jar was estimated to be around 3540 Pa, which is the sum of the pressure of the loaded weight (1540 Pa) and the pressure in the desiccator (2000 Pa). This value is reasonable considering that degassing effectively occurs at just above the vapor pressure of water. After the initial vigorous degassing, the speed slowed down, and discernible degassing was no longer observed; however, the process was allowed to continue for a further 15 min to ensure that as much dissolved oxygen as possible was removed. The degassing process was highly reproducible, and bumping of the monomer solution containing the surfactant (Tween 20) did not occur, even though the pressure of the vacuum pump was not regulated. Figure 2 shows the period of vacuum degassing necessary for the graft reaction (Reaction time: 3 h). Before degassing (1.5 min), the graft reaction hardly occurred. Degassing using a vacuum for 15 min was enough for the subsequent graft reaction.

**Figure 3:**
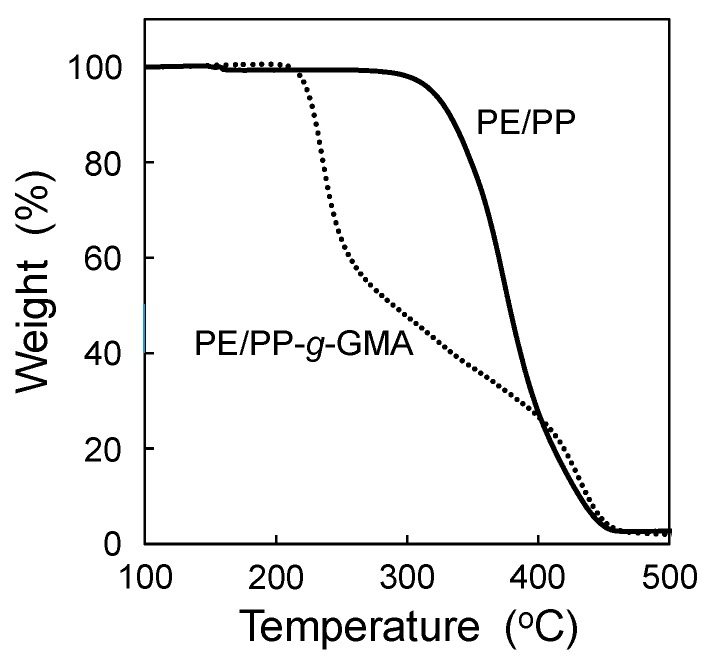
TGA curves for the PE/PP and PE/PP-*g*-GMA fabric under nitrogen.

The radiation-induced graft polymerization of a GMA monomer onto PE/PP fabrics using the SREG method was confirmed using not only gravimetric analysis but also thermogravimetric analysis (TGA) (Figure 3). As expected from the previous study [28], PE/PP-*g*-GMA fabric showed a fairly low decomposition initiation temperature (190 °C) compared with PE/PP fabric. This result indicates the introduction of GMA graft chains. The degree of grafting (*Dg* = 102%) calculated using the TGA shows quite good agreement with that using the gravimetric analysis (*Dg* = 106%). Accordingly, the SREG method could proceed like the conventional graft polymerization method by using a gas/vacuum manifold.

**Figure 4:**
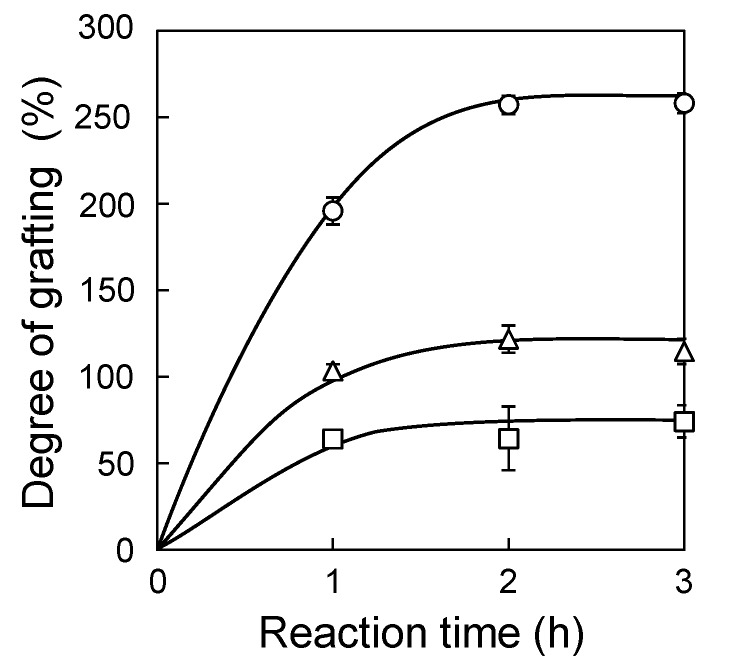
Graft polymerization using the SREG method at 20 (○), 10 (△), and 5 (□) kGy. Error bars indicate standard deviation.

Graft polymerizations of GMA monomers to PE/PP fabrics, irradiated at a dose range of 5–20 kGy, were carried out using the SREG method while varying the time of the graft reaction (Figure 4). Even at a dose as low as 5 kGy, the graft reaction proceeded and the degree of grafting obtained had very high reproducibility. Because the total amount of generated radicals was proportional to the adsorbed dose at the same irradiation condition, the initial velocity of grafting should be proportional to the dose of preirradiation. However, under 20 kGy, this was often not true using the conventional graft polymerization method via degassing using a gas/vacuum manifold. It is known that very low dissolved oxygen (<0.5 mg/L) inhibits the graft reaction under 20 kGy [29]. This indicates that the elimination of dissolved oxygen was not sufficient or oxygen had redissolved into the system. Using the SREG method, the initial velocity of the degree of grafting was 4.2, 2.0, and 1.1%/min at 20, 10, and 5 kGy, respectively. This result indicates that oxygen was sufficiently eliminated from the monomer solution such that the remaining oxygen had little effect on the graft reaction, even at 5 kGy. It is surprising that this easy method produced the initial velocity of grafting that was ideally proportional to the dose of radiation.

To accelerate development of graft material, a high-throughput screening method was required. It is difficult to obtain a large number of accurate and reproducible data points using the conventional graft polymerization method with a gas/vacuum manifold because of the complicated process. However, using the SREG method, it is easy to deal with a number of samples at the same time and at a small scale. Thus, this method is well suited to be a high-throughput screening for the graft reaction, which needs to include a simple and easy degassing process for the monomer solution. The SREG method can also be applied to other graft polymerizations by changing the loading weight to correspond to the vapor pressure of the reaction solvent.

### 3.2. Fabrication of Heavy Metal Adsorbent Using the SREG Method

Contamination of water by heavy metals is a serious threat to human health and the health of the environment, and heavy metal adsorbents having a high enough quantity of a chelate structure on the surface are, therefore, in great demand. In heavy metal adsorbents, the capacity of the adsorbent increases with an increase in the degree of grafting; however, the adsorbents become less flexible. Especially, over a 200% grafting degree, the adsorbents become brittle [30]. In other words, the adsorbent capacity is inversely correlated with the mechanical strength of the adsorbent. Therefore, the grafting degree of a heavy metal adsorbent has to be controlled to be over 50% and under 200% of the grating degree. The SREG method satisfies the grafting degree due to the easier irradiation at low absorbed doses that is expected to be applicable to the fabrication of heavy metal adsorbents. Its viability in synthesizing an appropriate heavy metal adsorbent was examined.

**Figure 5:**
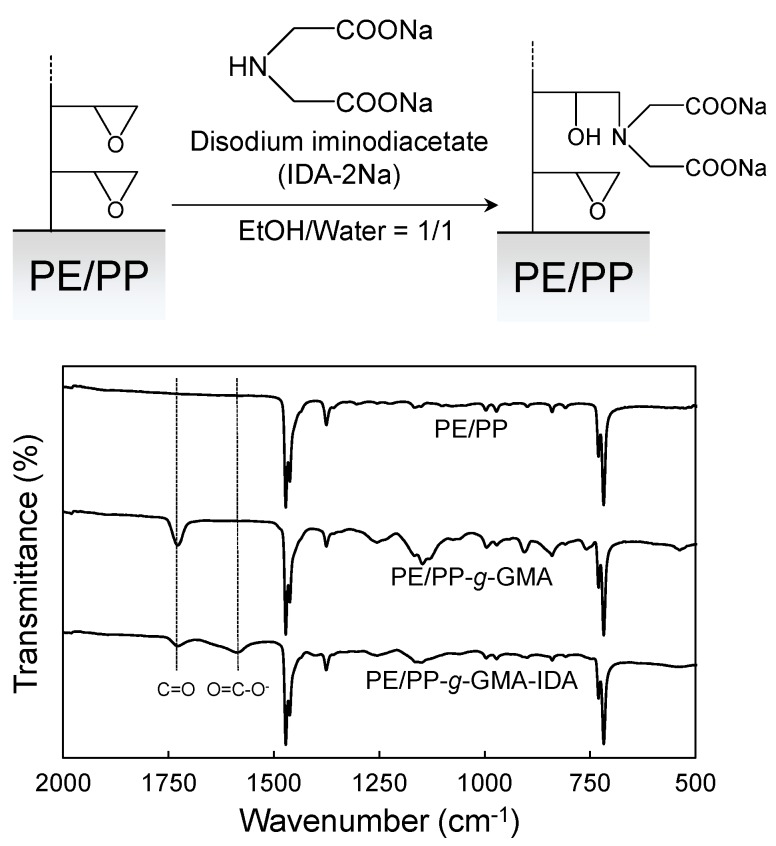
FTIR-ATR spectra of the PE/PP, PE/PP-*g*-GMA, and PE/PP-*g*-GMA-IDA fabrics.

After gamma-ray irradiation of the PE/PP fabric at a dose of 5 kGy, GMA was grafted onto the PE/PP fabric to produce PE/PP-*g*-GMA fabric using the SREG method (*Dg* = 72%). After the graft polymerization, the epoxy group of the GMA graft side-chain of the PE/PP fabric was converted to an IDA group to form PE/PP-*g*-GMA-IDA fabric. The IDA functional group is known to remove various kinds of heavy metals including Co(II) from wastewaters, and have been widely used as a commercial adsorbent [30,31,32,33,34,35,36,37,38,39]. To confirm the chemical changes produced by these successive reactions, the FTIR-ATR spectra of these fabrics were measured (Figure 5). After the GMA graft polymerization, an additional stretch at 1720 cm^−1^ was observed and assigned to the C=O stretching vibration of the ester in GMA [25]. The conversion reaction of epoxides to IDA gave characteristic bands in the 1570–1650 cm^−1^ region, which were attributed to the carboxylate salts of IDA [40]. These spectra indicated that the IDA group was successfully introduced onto the surface of the PE/PP fabric. The IDA group density and conversion were 1.6 mmol/g and 74%, respectively. In previous studies, the IDA group density of the adsorbent was mainly between 0.7 and 3.2 mmol/g, depending on the grafting degree and conversion [30,41,42,43]. The SREG method at low absorbed doses was expected to be useful to fabricate heavy metal adsorbent.

**Figure 6:**
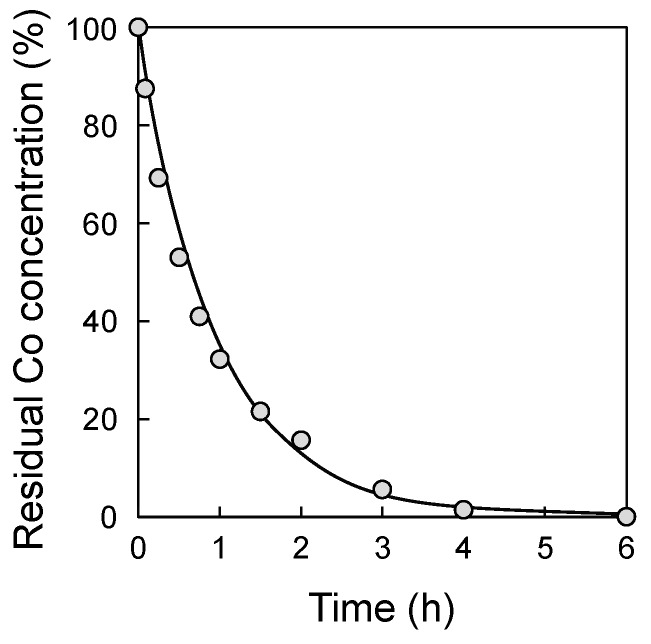
Co concentration (50 ppm) decay in water during the adsorption of Co(II) on PE/PP-*g*-GMA-IDA fabric.

The adsorption ability of the PE/PP-*g*-GMA-IDA fabric was evaluated using the batch adsorption test. The Co(II) ion was chosen as a model of a harmful heavy metal ion. Once Co(II) was adsorbed by the PE/PP-*g*-GMA-IDA fabric, the remaining concentration of Co(II) ion was determined using ICP-OES (Figure 6). The initial concentration of the Co(II) ion was 50 ppm, and the color of the solution was light pink. Over time, the color of the light pink solution became gradually lighter, and the Co(II) concentration reached the limit of detection within 6 h. This result indicates that the PE/PP-*g*-GMA-IDA fabric had enough adsorption ability to essentially remove Co(II) from a highly concentrated Co(II) solution.

Using the SREG method, (i) a facility capable of generating high-dose-rate gamma-ray radiation or (ii) expensive experimental equipment are not necessary to fabricate metal adsorbent materials. Recently, several studies about the grafting of functional molecules on natural fibers have been reported, especially in developing countries [44,45,46,47,48,49]. The natural fibers need low radiation doses because degradation of the cellulose, the main component of natural fibers, can remain limited in this range of radiation dose. The SREG method at a low dose is expected to be useful in the developing countries, which do not have high-dose-rate gamma-ray radiation or expensive experimental equipment. Moreover, scaling up the process is easy, and the method is expected to be successfully applied to the mass production of graft polymers.

## 4. Conclusions

The SREG method was developed using a commercially available sealed glass jar instead of expensive experimental equipment. Enough oxygen was removed from the monomer solution without bumping such that even at a low dose (5 kGy), the grafting reaction proceeded as expected. Compared with conventional graft polymerization methods that require a gas/vacuum manifold, the SREG method is very simple and provides highly reproducible data that can be used for high-throughput screening of the grafting reaction. The method was used to fabricate a heavy metal adsorbent, the PE/PP-*g*-GMA-IDA fabric, which demonstrated its ability to adsorb highly concentrated Co(II) ions down to the limit of detection using ICP-OES. Several samples can be produced using this method, and the reaction scale can be adjusted as necessary owing to the simple mechanism and procedure. Thus, the method will be valuable not only for the fabrication of functional materials produced using graft polymerization on an industrial scale, but also in high-throughput screening research of the graft reaction.
